# Extracts

**Published:** 1881-10

**Authors:** 


### Extracts.

Extripation of Ovaries—Report of Three Cases.—Case I is that of a virgin, aged twenty years, white, a resident of an adjoining State. Catamenial phenomena first appeared at the eleventh year, were normal, and so continued until August, 1876, her age being then thirteen years, at which time, and while much worn from long watching at the bedside of her sick mother, dysmenorrhœa was for the first time manifested, and of such severity as compelled her to remain in bed for several days.

For eight months she had been confined to bed, with constant pain in both ovaries and lumbar region of spinal cord. All her symptoms were greatly increased in severity at the time for each monthly flow; and for many months the actual flow had only taken place on each alternate period—dysmenorrhœa alternating with painful amenorrhœa.

Rational therapeutics having been exhausted without benefit to the patient, in consultation with Drs. Willett, W. E. Rogers, Sim, Wise, Overall and Sinclair, it was decided to remove both organs, because they were believed to be in a state of chronic inflammation, from which general peritonitis was liable to arise at any time. Secondly, chronic ovaritis is an acknowledged factor in the development of ovarian cysts. Thirdly, serious lesions of the nervous system were threatening. And lastly, the young lady was bed-ridden, with morphine habit engrafted, a burden to herself and family and so long as the organs remained in situ her condition promised only to grow worse.

Assisted by the above named counselors, and Drs. Laurence and Jones, on the 19th of February, '81, I removed both ovaries. No unpleasant symptoms followed the operation. The chart shows on the eve of the third day, a temperature of 100½°; next morning 98½°. On the eve of the sixth day it arose to 102°, but fell the following morn to

99° ; the eve of the seventh day marked 101°, while the morn of the eighth, and for ten days thereafter, her tem. perature did not exceed 99°. Bowels were moved with the aid of enema about the twentieth day. Very little local inflammation occurred, and at the end of the third week she was pronounced convalescent, from the operation.

*Ovaries.*—The left ovary was found to be soft and greatly atrophied, not being more than one half the normal size. In it was found all of the evidences of long standing oophoritis, involving both the connective and the follicular tissue. At the time of its removal a monocyst was ruptured, which projected about twenty-eight millimeters from the upper and external surface of the organ, and dipping down the entire depth of the parenchymatous, its base rested upon the medullary substance of the ovary. Thus making a cystic tumor, conical in shape, and measuring about twelve millimeters in diameter at the base in the ovarian stroma, and about thirty-three millimeters in its entire length. The contents of this sac, as observed previous to its rupture, was perfectly clear and limpid, while a beautiful net-work of enlarged capilliary blood-vessels covered its entire surface. But few Graafian vesicles were perceptible to the unaided eye; these were located near the uterine side of the cortical structure; they were uniform in size, and about as large as a small pea. Each appeared to have been arrested in its growth, and all contained a brownish, turbid fluid. The right ovary was normal in size and firmness, the Graafian follicles were of various sizes, from that of near approaching maturation to the smallest visible vesicle. Their contents was not, however, clear, as in the normal follicle, but presented an opaque, milky appearance, evidently from broken down cells of the membrana granulosa.

Case II. Jane W., colored, aged twenty-eight years. At thirteen she menstruated, and continued to do so, regularly and normally, until the third month after marriage, which took place in her twenty-second year. Six weeks after cessation of her menses she aborted.

Six years had elapsed since her trouble commenced, and in this period she had been variously treated for cystitis, metritis and displacement of the organ, but without benefit. Defecation was very painful. She was able to get about, but totally unfit for work. Her urine was heavily loaded with phosphates, (probably the result of mal-assimilation, due to the use of opiates, or else to prolonged strain on nervous system caused by pain and loss of sleep); at times it contained blood and mucus. Exploring the bladder with a sound, I found the mucous lining very sensitive, but not indurated; its muscular coat was hypertrophied, but I failed to find any foreign body.

Upon making a digital examination, I found vaginismus, and in the cul de sac of Douglas detected what appeared to be an inflamed ovary. The examination caused so much pain that I was compelled to desist. During the following week I made several unsatisfactory examinations, and finally, with the aid of an anæsthetic, by rectal and vaginal exploration, found both ovaries displaced, and bound by adhesions in the cul de sac of Douglas; both were exceedingly sensitive to pressure. The uterus was normal in every particular.

On the 29th of April, 1881, with the above named and several other medical gentlemen assisting, I removed both ovaries.

Convalescence was very tedious, and ofttimes doubtful, owing to repeated imprudences on the part of the patient.

Eighty-one days after the operation she came to my office. I examined her and found vaginismus had entirely disappeared; there remained some induration, the remains of pelvic cellulitis, with a slight discharge of pus from the pelvic abscess. She passed water only five or six times in twenty-four hours, and was perfectly comfortable.

*Ovaries.*—On the upper surface of the right ovary there was observed a slight elevation somewhat discolored, and upon making a section through the center of the discoloration, a cavity occupying more than one-half of the organ was disclosed. Its contents was semi-fluid and of a dark chocolate color, the walls of the sac hypertrophied, and in the surrounding connective tissue, evidences of inflammatory action of long standing. There were but few Graafian follicles to be found in the remaining stroma; these were small and had undergone more or less cystic alteration. No corpora lutea.

The left ovary presented evidences of both follicular and interstitial inflammation, but in a less marked degree. Ovulation had evidently been going on in this, as two corpora lutea in different stages of retrogressive change were observed.

Case III. Miss Mollie R., white, virgin, age twenty-two. Menstruated at thirteen without difficulty. Nine months ago she sprang from a porch to the ground, a distance of nearly five feet, alighting on her heels, and jarring her considerably. Vaginismus existed, as in two previous cases. The uterus was in its proper position, of proper size, and in a healthy condition. Menstruation was perfectly regular and accompanied by an increase of pain in the left ovary alone. The right ovary was of the proper size, in proper position, and not abnormally sensitive. It was after quite a search Dr. Sim discovered the left ovary, very sensitive, and situate quite above and rather external to its normal position.

Assisted by the above named gentlemen, on July 21, 1881, I removed the left organ, which was found at least one inch and a half above and external to the usual site, and firmly bound by inflammatory adhesions. Its surface was rough, like the undressed side of sole-leather, while the organ itself had a soft, pulpy feel. The right organ was examined, and, presenting no evidence of disease whatever, was left in situ.

My patient made a most excellent recovery from the operation, and at the fourteenth day was considered out of danger. Her bowels were moved on the thirteenth day. At no time did temperature exceed 102°—most of the time below 100°. Time enough has not elapsed to say if benefit follows the operation. She sleeps well, suffers very little, but has not been allowed to attempt walking. From the condition of the ovary, as seen below, we have every ground for feeling confident that, the source of irritation being done away with, she will soon be entirely restored.

*Ovary.*—The ovary when removed was spherical in shape, measuring fully twenty-seven millimeters in diameter. On making a section of it, a quantity a clear fluid escaped, allowing the organ to somewhat resume its normal shape. It then measured twenty-six millimeters in length, ten in width and five in thickness. It had lost all of its elasticity and was quite soft. No corpora lutea could be found, and but few Graafian vesicles, the latter only partly filled by their contents.

Laparotomy was the method chosen in all three cases. In neither case did the incision through the integument exceed three and a half inches, while the peritoneal incisions were from two and one-fourth to two and three-fourth inches. Pedicles were tied with carbolized animal ligatures, and returned to the cavity. Incisions in each case were closed with three deep and three superficial silver sutures. Listerism strictly observed in cases I and III, but spray in case II became choked in midst of operation, which was completed without spray. The sutures were removed between the eighth and fourteenth days. In case I a few drops of pus followed removal of one of the deep sutures—no more. In case II a collection of pus took place between the muscles of the abdomen, discharging an ounce per day for over a week. This case was very unruly, and I am confident there would have been no collection of pus had she not sat up and exerted herself. In case III one drachm of pus formed around and discharged with the removal of one deep suture. A sanious discharge, a metrostaxis, came on in each case within a few days after the operation, and continued from five to fifteen days, coming later and disappearing sooner in case III. Cases I and II have, to date, experienced all the symptoms of ovulation save the actual flow, no trace of which has been seen since the cessation of the metrostaxis, mentioned above. In each case the operation was performed just after the menstrual flow, or just after the usual time therefor.—*W. B. Rogers, M.D., in Miss. Valley Medical Monthly.*

---

THE July number of the *North Carolina Medical Journal* publishes a paper entitled "Surgical Notes," by Robert Battey, M.D., Rome, Ga. The paper was intended for reading at the last meeting of the Medical Association of Georgia, but was not presented, owing to the absence of the author. He presents what the manufacturers have christened "Battey's Universal Syphon Catheter." It is the ordinary soft rubber catheter of Nelaton, extended to a length of four feet. Its syphon action, as well as its other advantages, will be at once appreciated for use in male or female.

"A New Perineal Retractor" is also described. This is a modification of Sims' speculum. The blade is shorter and less cupped than in Sims' ordinary instrument. Some other changes are noted. He claims that it better serves its purpose in operations upon the uterus and vagina, as well as in the more common manipulations upon those organs.

To the paper, Dr. Battey adds a note on surgeon's sponges. A new sponge is objectionable on account of the grit and calcareous matter it contains. He therefore prefers one that has been repeatedly used, but that has been cleansed by maceration in a solution of one ounce of aqua ammonia to one quart of water; this to be methodically and persistently done. A number of experiments seem to demonstrate that the sponge is well cleaned and disinfected by this process. This agent dissolves and removes the albumen of the blood, while carbolic acid, more generally in use, hardens and fixes such substances in the meshes of the sponge.

---

Animal Magnetism—Mesmerism, which has long been the sole property of charlatans and spiritualists, has lately been receiving the careful attention of neurologists of the old as well as the new world.

Let us examine what occurs in the process of hypnotizing. An individual may be taken who is quite sensitive to the process. He looks steadily into the eyes of the magnetizer, while the latter makes slow movements with his hands from brow to chin, a short distance from the surface. The eyelids of the subject become heavy, they twitch for a short time and finally close. That patient is hypnotized. If we have a less sensitive subject to deal with, it may be necessary to prepare him by causing him to look upon some bright object close to his eyes. In doing this the retina becomes exhausted, and with it the brain substance. Another subject may be so sensitive that looking at an inanimate object suffices to put him in the hypnotic condition. Another still may be mesmerized by the ticking of a watch. All the sensory irritants that act as artificial hypnotics have one quality in common—they are feeble, continuous and monotonous. When the hand is slowly moved over the vicinity of the face it acts as a slight irritant to the delicate sensory nerves, through the difference in warmth and moisture, as well as through the feeble current of air produced. The reason that one individual can hypnotize when another cannot, depends, not upon any magnetic fluid that the other does not possess, but rather upon differences in temperature and humidity of the hands, as well as upon the manner in which they are passed over the face, and the belief in his power entertained by the subject.

For the purposes of this investigation, we may look upon the brain as consisting essentially of three parts; a thinking surface, the organ of consciousness, a sensory centre, to which the irritations of the peripheral nerves are telegraphed, and a motor centre, from whence the muscles receive their commands.

In the hypnotic condition, by the action of the long continuous feeble irritants we have employed, the thinking part of the brain has been particularly placed out of function. The resistance between the sensory centre of consciousness as well as between the latter and the motor centre, has been increased; whereas, that between the sensory and the motor centres has been diminished. Hence, when an irritation is conveyed in a hypnotized individual to the sensory centre, either from the skin, or the eye, or the ear, it passes directly to the motor centre and produces the effect.

I have here one of your young fellow students, Mr. A. I make a few passes over his face, and, being an excellent subject, he soon becomes hypnotized. I tell him that I have a pear in my hand, and ask him what it is, showing him this potato. You hear he answers it is a pear. I tell him to eat, and with evident relish he masticates it. I now tell him it is a piece of a cadaver, and instantly you see the potato thrown away, and the expression of pleasure evident a moment ago changes into intense disgust. He acts exactly as one would who had by accident placed a nauseous substance in his mouth. I tell him it is sugar; he smacks his lips, and when I ask him how it tastes, he tells us sweet. I place this rubber on the desk and tell him it is a baby; he must steal it. He makes the appropriate stealthy movements and seizes it. I ask him, then, if that is the way to hold a baby. He answers no, and folds it in his arms as a mother would her child. I ask: "What do you want to do with the baby?" "I do not know," he answers. I suggest that he desires to dissect it; he acquiesces. You see the subject has reacted to every suggestion made to him.

We know that if the pneumogastric nerve be irritated the action of the heart ceases, and if the superior laryngeal nerve be irritated respiration ceases. It may be that a similar depressing action is exerted upon the gray matter of the brain by the feeble irritants we have described. Is it expectant attention alone that produces the wonderful effects you have observed, as is maintained by Carpenter? By no means. I have here a little patient in whom none of you will suspect either expectant attention or collusion. It is the pigeon I hold in my hand. I hold him a few moments, and you see him bedazed. I turn him on his back—he is indifferent.

I take a frog and hold him for a few moments, and now, although before it was impossible to induce the animal to remain on his back, you see I can turn him as I choose without any resistance on his part. I move his extremities into any position—they remain there. The frog is in a cataleptic condition, and shows most distinctly the characteristic waxy flexibility.

When I first took them in my hand you observed how they struggled; fear possessed their minds in consequence of the sudden irritation. No unpleasant sensation following, fear subsided, and all that acted upon them was the feeble irritant—the warmth of my hand applied to their skin. This feeble, monotonous irritation conducted to the brain has produced that peculiar change, in precisely what manner it is true we know not, that we have already described. The passage way between the sensory and motor centres has been made easy, whereas the resistances on the road to the thinking brain surface have been increased.

I hope, gentlemen, it has been clear to you by the preceding remarks that in mesmerism we have merely the effects of long continued, feeble and monotonous irritation, assisted certainly but not necessarily by expectant attention; that it is merely a physiological fact, no misticism, no animal magnetism, no otic fluid, no humbug.—*J. O. Hirschfielder, M.D., in Pacific Med. and Surg. Journal.*

---

ABSTRACT OF A CLINICAL LECTURE BY DR. WILSON FOX.—The doctor, exposing her neck, pointed to some deep, puckered scars, and then proceeded to discuss the case, as I shall now most imperfectly imitate:

"These, gentlemen, are the indications of what were, and are, called 'scrofulous glands.' There has been a great deal of discussion as to the real nature of the changes in the glands so affected; but at present the changes are generally considered to be of a tuberculous character. In this young girl's lung there is a large cavity; if you listen behind, or, better still, low in front, on the right side, you will hear large moist sounds, and what is commonly called cavernous breathing. In this case it is rather more like large tubular breathing. This is not a case of tuberculous phthisis—that is to say, not primarily so; there may now be, or may be at a later period, tubercles scattered through the lung; but the character of the onset, the diathesis of the patient, the extent of the disease, and the immunity of the other lung, all point to 'pneumonic phthisis' as the form of disease here present. Now let us consider the sputa; it was abundant, purulent, and very fetid. This points either to gangrene, bronchial dilatation, septic processes due to germs, or to a very strong preponderance of the destructive over the formative changes in the body. To the latter, we must, in this case, largely attribute the fetor. You observe, too, that this patient has had a strong tendency to sweating, which also points to the same destructive changes I have referred to. The temperature in this case is most instructive, and is of the utmost importance in the diagnosis. You will observe from this chart (a large and very fine one it was) that for weeks there has been constant pyrexia—the temperature really may be said never to have been below 100°, and generally it has been above that. It shows, it is true, considerable oscillation, but still, be it marked, there is constant fever. This indicates that the disease is pursuing an acute course, there is rapid disintegration of tissue, there are constantly preponderating disintegrating changes. Note, also, the delicate aspect of this patient—the thin skin, light, fine hair, etc. But you who have been following the case will admit there has been very considerable improvement; and this brings me to the very important matter of the treatment of this class of cases. The sputa now are no longer fetid. What have we done? We have used inhalations of a disinfecting material (one of the oils) in this, the best thing I know of for the purpose—'Wordsworth's washable respirator,' which, as it can be so easily washed, can be itself kept perfectly clean. But there are other modes of disinfecting the sputa in the air passages. One is to have an atmosphere more or less saturated with carbolic acid, another, to allow iodine to evaporate in the patient's room. But it will not do to check expectoration —such a result would be disastrous, as increasing the very condition we wish to obviate. The next point to be attained is to lessen perspiration, or, rather, to meet the condition which gives rise to it. In getting rid of the fetor of the sputa, we have contributed to this in part already; but as sweating arises when the arteries are dilated, the tension diminished, and, therefore, occurs during sleep, it is obvious we should look for remedies that tend to counteract this. To this class belong belladonna, picrotoxin. etc. Oxide of zinc does good in a large number of cases, though I cannot explain its action. But one of the best remedies is to give the patient alcohol at *the critical time.*

If such patients would wake up in the small hours of the night and take a dose of alcohol they would be saved much discomfort. But here, again, one is met by a theoretical difficulty, for alcohol is considered a dilator of vessels. But in treatment of cases like this before us, there is nothing so important as to diminish pyrexia ; so long as this continues, the patient's course will be retrogressive. In the first place, absolute rest in bed must be strictly enforced ; the utmost possible tranquillity of mind and body must be enjoined ; all movement, so far as possible, must be forbidden. This having been done, we are lead to ask, What can be accomplished by medicine? And in this matter, gentlemen, we must simply confess our failure and our ignorance. One remedy after another has been vaunted ; I have tried them all, and I must say there is no remedy or medicine that will permanently reduce the pyrexia. You may give a large dose of quinine to-day, and find the temperature down to-morrow ; but you can never be certain that it will not rise again, you can never know when it may rise, and all the while your patient is in other respects subjected, it may be, to much discomfort in consequence of our futile medication ; and the same applies to the other so-called antipyretics. You may do something by dieting. Give light, easily digested material. If the pulse is rapid, you may give aconite, or, perhaps better digitalis, which also, in a certain proportion of cases diminishes the sweating. But among our most usefu remedies to lower temperature, induce tranquillity, and prevent tissue waste, etc., is opium ; not in doses to induce sleep at night—this is a wretched practice—but in small doses. When opium checks expectoration unduly, or causes sweating, it, of course, must not be given. But, gentlemen, when you commence to give opium in cases of this kind, as it may be necessary to continue it for some time, you incur a grave responsibility. Remember, you may forge for your patient a most galling chain ; for there is no habit that men even of the strongest wills find it so hard to conquer as the habitual use of opium. Take care that you always be sure that at any stage your patient can leave it off. But in cases of phthisis, there is another remedy you will find mentioned in your pharmacopœias, viz.: Vinum—wine. It is as much a medicine as opium or quinine. Much that I have said of opium, however, applies to it. It is true we sell opium to the natives of the East, by which traffic we rendered it necessary that laws should be enacted to limit its use to prevent the actual extermination of the inhabitants of some of those countries; we say this 'to the great glory of the British nation.' But it is to no such abuse of a valuable drug I am now referring, but to alcohol. The distinguished Dr. Flint, of America, has abundantly shown the great value of alcohol in certain cases of phthisis. Now it is in just such cases as this it is useful. But be careful, as of opium, that your patient can leave it off at any time, and always prescribe alcohol in definite doses. Do not allow it to be slopped about in tumblers, for people often then give it 'as if they loved you.'

"'A great deal has been said of climate; but in such cases as this one is, you must not put much dependence upon it. It is possible that there may be isolated cases, in which, with even a temperature of 100°, it may be worth while allowing a patient who has plenty of money, and has set his mind upon a journey, to take it; but for patients with high temperature, as a rule, any sort of move is extremely hazardous. It is better to risk a patient even in a climate such as ours, than to sanction any sort of journey with its excitements of body and mind. To talk to tne poor of the advantages of climate is simply to be guilty of cruelty."—*T. W. Mills, M.D., in Canada Med. and Surg. Journal.*

---

Treatment of Naso Pharyngeal Catarrh.—In the discussion of the much-discussed subject of naso pharyngeal catarrh, there seems to me to have been a lack of precise statement of the indications for treatment. Different procedures have been recommended with the general and vague object of suppressing the discharge, without a definite statement of the manner in which they are expected to contribute to that result. A step in advance will have been made when we have an exact idea of what is to be done, so that we may select the best method of doing it.

The indications for treatment, it appears to me, may be concisely formulated as follows:

*First.*—Keep the parts clean.

*Second.*—Remove all sources of irritation resulting from occupation, residence, climate, etc.

*Third.*—Enforce attention to hygiene and to the general health.

*Fourth.*—If there is obstruction to nasal breathing, remove the obstruction.

*Fifth.*—If there is ulceration, use strictly local applications, with a view to healing.

*Sixth.*—If there is hypertrophied glandular structure yielding excessive secretion, remove the hypertrophied portion.

*Seventh.*—If, after the above indications have been fulfilled, there is still hyperæmia, use mild astringents and sedatives, and such constitutional treatment as is thought to be indicated.

*Eighth.*—Cease treatment the moment there is no longer a definite indication for it.

As to the first three indications, the reason for them is too obvious to require comment.

The fourth indication, relating to obstruction to nasal breathing, is of very great importance, chiefly, as I think, for a reason which I believe has not heretofore been brought forward. I refer to the effect of nasal stenosis in causing hyperæmia of the membrane behind it, owing to the rarefaction of the air with each inspiration. The influence of this diminished air-pressure behind the obstruction upon the development of the bony and cartilaginous structure of the nose in early life has lately been insisted upon by Dr. Roe, but he has overlooked the fact that all the tissues subjected to the action of this partial vacuum must suffer a proportionate congestion as certainly as the skin under a cupping-glass. From congestion to hypersecretion is but a single step, and here I think we have a key, not only to the persistence of the discharge in many cases, but to the prompt abatement of it which often follows relief of the stenosis.

The fifth indication, relating to direct applications to ulcerated points when practicable, should discourage the use of strong irritating solutions, sprays, or powders to the whole mucous surface, in order to heal ulcerations of a limited portion of it.

The sixth indication, as to hypertrophied glandular tissue, requires a discrimination between that which produces hypersecretion and that which does not. The former should always be removed, the latter never unless it obstructs nasal breathing, which would bring it under the fourth head.

If the preceding indications have been fulfilled, the one relating to the use of astringents and sedatives will seldom be present for any lengthened period. Still their use for a limited time will often hasten the return of the vessels to their normal calibre. As to the value of constitutional treatment in non-specific cases, nothing is as yet definitely settled.

The last indication is far from being the least important. Continuing local random treatment may perpetuate the disease indefinitely, and when the cure has reached a point at which we no longer know just what we expect our remedies to do, it is time to lay them aside and give nature an opportunity to complete the work.—*Andrew H. Smith, in N. Y. Med. Record.*

---

Treatment of Aural Cases.—In every patient complaining of defective hearing, the external auditory meatus should be always explored, and to its very bottom. The drumhead is recognized as a whitish membrane, located about one and one-fourth inch from the outer orifice of the ear. In a proper light a prominent white line is seen coming from above and extending vertically downwards to the centre of the drum membrane. This white line is the long process of the malleus imbedded in the substance of the drumhead. From the free extremity of this bony process, extending obliquely downward and forward, is a triangular bright spot of reflected light, which when seen means always a clear and unobstructed external meatus.

If all the ceruminous deposits were as black as those seen in the ear of our dispensary patients there would never be difficulty in detecting them. There are ear plugs of a much paler hue, approaching even to a dirty white color, depending upon incorporation with an amount of exfoliated epidermis from the skin lining the passage. When such a small whitish plug lies deep in the ear, it may readily escape detection by the inexperienced in aural examinations. If we, however, have fixed in our minds, that the bright spot of light on the drumhead at the extremity of the bony process, imbedded in the centre of the drum membrane, is an important factor in a successful examination, we then have positive data for our explorations. See this bright spot and you will then feel assured that no wax accumulation of an annoying character can be present.—*Julian J. Chisolm, M D., in Maryland Med. Journal.*

---

AN EXPERIMENTAL INQUIRY INTO HUMAN MILK, AND THE EFFECTS OF DRUGS DURING LACTATION ON EITHER NURSE OR NURSLING —Mr. Thomas M. Dolan and Mr. W. H. Wood commence in the *Practitioner* a series of interesting papers on the above subject, undertaken by Mr. Dolan in order to answer the frequent question: "Will my medicine have any effect upon the child?" That medicines and food do affect the milk, and consequently the child, is certain. Frequently, as Mr. Dolan shows, a wet nurse on her first arrival, causes the child to thrive; but soon all becomes changed, because, from a laborious out-door life, with few luxuries, the woman is surrounded with good living, and has very little exercise, and consequently her milk ceases to nourish the child. Cause her to resume, as nearly as possible, her former mode of life and food, then her nursling quickly recovers. A clear description of the best method to analyze human milk is then given, which is, however, too long for insertion, and cannot be condensed. The practical outcome of these elaborate papers is summed up as follows: 1. All therapeutical agents intended to act on the mammary gland must first enter the blood. 2. All drugs derived from the natural orders, lilacæ, cruciferæ, solanaceæ, umbelliferæ, etc., enter the blood and impregnate the milk; hence caution is needed in giving such drugs to nursing women. 3. The only approach to a true galactagogue is jaborandi. 4. Belladonna is an antigalactagogue. 5. In inaction of the mammæ, the milk may be increased and influenced by medicines. 6. The milk of the mother may be increased in heat-forming elements by the administration of fats. 7. The salts of milk may be improved by the administration of medicines. 8. Various physiological actions—purgative, alterative, diuretic, etc., may be produced in the child by administering drugs to the mother. 9. If we are to expect any improvement in milk-secreting power, both as to quantity and quality, we must look to diet for the attainment of that object.—*Richard Neale, M.D., in London Medical Record.—N. C. Med. Journal.*

---

INSANITY AND CRIME.—That there are insane criminals there can be no doubt. The important question with this class of cases is, what shall be done with them? We have long been convinced as to what the proper course is to adopt in such cases. It is this: Whenever, in the case of murder or any other flagrant crime against society, the plea of insanity is set up and successfully maintained, then the penalty should be the incarceration of the criminal for life in a prison asylum. Under no circumstances should such a person be turned loose into society again, after having manifested such dangerous tendencies.—*Journal of Nervous and Mental Diseases.*

---

BROMIDE OF ETHYL.—The following are the conclusions deduced by MM. Bourneville and H. d'Olier from a series of researches on the physiological and therapeutic effects of bromide of ethyl, published in the *Progres Medical.*

1. The pupillary dilatation at the beginning of the inhalation of the bromide of ethyl is not at all constant.

2. Complete muscular resolution is the exception.

3. The anæsthesia produced varies to a large degree in different subjects.

4. The temperature, the secretions, and the general condition appear to undergo no modifications.

5. The pulse and the respiration are slightly accelerated.

6. A tremor, more or less pronounced, of the members may be produced during the inhalation, but it does not persist beyond this.

7. Hysterical attacks are generally easily arrested by the bromide of ethyl.

8. Epileptic attacks may sometimes be cut short by giving the drug during the tonic period, but more frequently the inhalations are ineffectual.

9. In epilepsy the regular employment of bromide of ethyl, administered in daily inhalations during a period of two months, notably diminished the frequency of the attacks.—*Journal of Nervous and Mental Diseases.*

---

Excision of the Stomach.—A most remarkable case of surgery by Professor Billroth, who, it will be remembered, reported two cases of œsophagotomy—one of them successful—July, 1881.

On the 29th ultimo, Prof. Billroth, of Vienna, excised about six inches of the greater curvature of the stomach, including the pylorus, for infiltrating carcinoma. The patient was a woman, aged 43, mother of eight living children, who was suddenly seized with grumous vomiting last October. Marked symptoms of cancer of the stomach and stenosis speedily followed. The operation was decided on in consequence of incessant and uncontrollable vomiting which threatened inanition. The operation lasted one hour and a half. There were extensive adhesions to the omentum and colon. Fifty silk sutures were used to unite the duodenum and the remaining portion of the stomach. On the fifth inst., (a week after the operation) the sutures were removed from the external wound, which had united without reaction. The patient was doing well, and was able to take coffee, tea, and other light nourishment. This is the second time the operation has been done. D. Pean did it in 1879, removed a carcinomatous pylorus. In that case catgut ligatures were employed. The patient died on the fourth day.—*Lancet.—N. C. Med. Journal.*

Prof. Billroth's patient also died.

Strapping the Breast in Mastitis.—L. S. Oppenheimer commends most highly the practice of strapping the breast in inflammation of that organ. He says:

"I have been unable to discover the name of the originator of this method of strapping the breast in mastitis, but his name certainly deserves to be written high as a benefactor to women. By the early compression with adhesive straps not a single case of mastitis in my practice has ever terminated in abscess. No matter how agonizing the pain, it is relieved quite as quickly as it would be with a hypodermic injection of morphia."—*E. M. Nelson, M.D., in Ind. Med. Rep.—St. Louis Courier of Medicine.*

If we remember correctly, Prof. L. A. Dugas, of Augusta, Ga., can afford some information on the subject.

---

Unnecessary Operations in the Treatment of Diseases of Women.—Dr. Clifton E. Wing, of Boston, has written a pamphlet with the above title, which gives the key-note to the reactionary current which is setting in against a large number of the surgical operations now, and formerly, in vogue for the cure of some of the diseases of women.

Dr. Wing has taken the following not unusual gynecological procedures, to illustrate his position:

(1.) The operation for ruptured perinæum. (2.) Division of the neck of the womb. (3.) The operation for restoring the neck of the womb where this has been torn (Emmett's operation). (4.) Curetting of the uterine cavity. (5.) The operation upon the anterior vaginal wall for prolapsus of this portion and cystocele.—*N. C. Med. Journal.*

Reactions often go too far, though well enough in their place. Operations may be unnecessary in some cases where made; but this does not argue that they are always unnecessary operations.

---

Testing Clinical Thermometers.—In accordance with the terms of the circular the Observatory has issued 1667 certificates with thermometers designed for physicians' use and physiological research. This great improvement is strikingly illustrated by the fact that while in June, 1880, four-fifths of all the thermometers received (representing seven different makers) were in error over one-third of a degree, and two per cent. had errors exceeding a whole degree, in April and May, 1881, four-fifths of all the thermometers sent had errors less than three-tenths of one degree. It is with feelings of surprise, however, that we have received in the course of the year, perhaps, fifty thermometers taken from active practice whose errors exceeded a degree and a half. There have been, comparatively, but few physicians' thermometers made in this country which have united accurate graduation of the scale with the requisite age of tube necessary to preclude further sensible changes, and there is little doubt that the great majority of physicians' thermometers now in use in the United States are from one-half to two degrees too high in their indications.—*Report Winchester Observatory.*

---

Advance in Therapeutics in 1880.—New remedies many, a few good, many bad, most indifferent. Tonga valuable in facial neuralgia; sulphide of calcium in suppuration—its action marked and reliable, grain doses now admitted; the nitrites of potassium and sodium have the action of amyl nitrite, but milder; ergot (again?) found useful in diabetes; pilocarpin useless in hydrophobia, which still defies all treatment; this last drug, tried in many directions, gave mêagre results; benzoate of sodium commended in scarlet fever and gonorrhœal ophthalmia; salicylate of sodium, according to Dr. Greenhow, mitigates but little the complications of rheumatic fever, while it may be a positive injury to the heart; salicin is inefficacious, while salicylate of quinia is highly praised by Dr. Hewan; the value of cold baths in typhiod fever have become more than doubtful.—*Chicago Med. Journ. and Exam.*

---

In a clinical lecture on Bright's disease, by Dr. Wm. Pepper, are found the following concluding paragraphs:

We will also try to free his system from effete matters, by the use of jaborandi. Last Sunday he received six doses, of ten drops each, of the fluid extract of jaborandi. That evening he sweated quite freely. On the 18th, Monday, his œdema having decreased then, one-half drachm of jaborandi leaves were put in four ounces of hot water and strained, and the liquid thrown into the rectum. This operation occasioned a most profuse sweat. This morning he is a little brighter. Since Saturday night he has had no tendency to convulsions. We will wait until to-morrow, when we will give him another dose of the infusion.

We will administer to him the bichloride of mercury, with the tincture of the chloride of iron, as soon as he is in condition. This treatment is exceedingly good, particularly where there is a tendency to congestion. We will give one grain in two ounces of the tincture, and of this mixture we will at first give 10 gtt. or 1-120 grain of the bichloride; this is a very small dose, it may be said, but it must be remembered that the membranes are often very irritable. If he can tolerate 10 drops it will be increased to fifteen drops or 1-80 gr., and afterwards to twenty drops or 1-60 of a grain. When we have once succeeded in giving twenty drops we will stay there and not try to increase it any more. It is to be given after food, three times a day. His only food will be skimmed milk, as it has been; the quantity, however, will be gradually increased. We will, beside, give him the infusion of jaborandi every other day, until he is rid of the dropsy.—*Med. and Surg. Reporter.*

---

A French court has decided that promises made to a doctor by a sick person are not valid at law. The ground for this is that the patient is no longer master of his will, and any agreement entered into must be under the influence of either fear or necessity.—*Lancet and Clinic.*

It does not require a French court to teach us, on this side of the water, that a sick man's promises often are as invalid as himself.

---

Diploma Selling Colleges.—The *Michigan Medical News*, remarks that the Mass. State Med. Society is making an effort to purge that state of quacks. To this end it has furnished to a legislative committee a list of medical colleges, regularly chartered, which confer diplomas without attendance or examination. These are: American University of Medicine and Surgery, Philadelphia; Philadelphia University of Medicine and Surgery; Physio-Eclectic Medical College, of Cincinnati; Physio-Medical College, of Cincinnati; American Eclectic Medical College, of Cincinnati; St. Louis Homœopathic Medical College; St. Louis Eclectic Medical College; New England University of Medicine and Surgery, of Manchester, N. H.; University of Medicine and Surgery, of Haddenfield, N. J.; and American Vitopathic College, of Cincinnati.—*N. O. Med. and Surg. Journal.*

---

Carbolic Acid Poisoning after Amputation of the Thigh.—This patient had been subjected to the operation of amputation of the thigh for ununited fracture of the femur. For five days after the operation the urine was green in color, an effect due to the absorption by the wound of carbolic acid, used in accordance with the antiseptic method of operating.—*H. B. Sands, M.D., in N. Y. Med. Record.*

---

The New Anæsthetic.—A correspondent of the *Virginia Medical Monthly* relates that some students (not medical) administered, by inhalation, two ounces of the *perfume* of the skunk to one of their fellows. Profound anæsthesia was produced, similar to that of the more ordinary agents.

Several thoughts present themselves. 1st. We would prefer some other anæsthetic, one differing both in name and odor. 2d. Was the "joke" upon the victim, who found refuge in sleep, or upon the jokers, who remained awake in company with the perfume? 3d. It must have required some effort and care to secure, in one vial, two ounces of this very diffusible agent.

